# Resources, challenges and way forward in rare mitochondrial diseases research

**DOI:** 10.12688/f1000research.6208.2

**Published:** 2015-08-13

**Authors:** Neeraj Kumar Rajput, Vipin Singh, Anshu Bhardwaj

**Affiliations:** 1 Open Source Drug Discovery (OSDD) Unit, Council of Scientific and Industrial Research, New Delhi, 110001, India; 2 Amity Institute of Biotechnology, Amity University, Noida, Uttar Pradesh, 201301, India

**Keywords:** rare disease, mitochondria, mitochondrial DNA, genome variation, crowdsourcing, crowdfunding, semantic-web, next generation sequencing

## Abstract

Over 300 million people are affected by about 7000 rare diseases globally. There are tremendous resource limitations and challenges in driving research and drug development for rare diseases. Hence, innovative approaches are needed to identify potential solutions. This review focuses on the resources developed over the past years for analysis of genome data towards understanding disease biology especially in the context of mitochondrial diseases, given that mitochondria are central to major cellular pathways and their dysfunction leads to a broad spectrum of diseases. Platforms for collaboration of research groups, clinicians and patients and the advantages of community collaborative efforts in addressing rare diseases are also discussed. The review also describes crowdsourcing and crowdfunding efforts in rare diseases research and how the upcoming initiatives for understanding disease biology including analyses of large number of genomes are also applicable to rare diseases.

## Introduction

Mitochondria are organelles present in every cell of the body (except red blood cells) and generate almost all of the energy needed by the cells to grow and sustain life. In addition to adenosine triphosphate (ATP) generation, mitochondria are involved in a large number of specialized functions in major cellular pathways including apoptosis, urea cycle, pyrimidine biosynthesis, heme synthesis, etc ^1^. The proteins that take part in these pathways are encoded both by mitochondrial DNA (mtDNA) and nuclear DNA (nDNA) ^2^. mtDNA encodes only a limited number of genes (37) which code for 13 proteins, two rRNAs and 22 tRNAs ^3^. These proteins code for four respiratory complexes of the oxidative phosphorylation (OXPHOS) system. The only non-coding segment of mtDNA is the displacement loop (D-loop, 1121 bp) that contains the origin of replication of the H-strand (OH) and the promoters for L and H-strand transcription. The diseases related to mitochondrial dysfunction are due to mutations in both the mtDNA and nDNA encoded components. Genetically, mitochondrial diseases are characterized as (i) those with sporadic or maternally inherited mtDNA mutations, (ii) those with abnormalities with Mendelian transmission of the trait, *i.e*., disorders believed to be due to mutations in nuclear genes that control mitochondrial biogenesis, and (iii) those that are caused by nuclear genes but are misinterpreted as mitochondrial based on the biochemical findings ^4, 5^. mtDNA point mutations which can either be maternally inherited or generated somatically have been associated with many diseases like A3243G for MELAS, A8344G for MERRF, T8993G for NARP, etc. Also, there appears to be a class of slightly deleterious mutations that modify the risks of developing certain complex diseases or trait ^6^.

Human mtDNA and nDNA mutations causing mitochondrial dysfunction are implicated in a broad spectrum of diseases affecting various tissues like brain, heart, liver, skeletal muscles, etc ^7^. The clinical symptoms of the disease depend on the cell type affected and range from loss of motor control, muscle weakness, cardiac disease to visual or hearing loss, etc. ^8^. Given that mitochondria are involved in a large number of cellular pathways, it is always challenging to correlate the exact causative role of genome variation with the observed phenotype. Additionally, the varying spectrum of disease symptoms is a major deterrent in early disease diagnosis. This is clear from the fact that with over 5000 mtDNA variation reported across databases, pathogenicity assignments for most of the variation is only limited to the association with the phenotype without any conclusive evidence on its causative role. While there are a large number of resources available on various aspects of human mtDNA, a major bottleneck is the lack of documentation of genomic variation data across populations with clinical details to evaluate these variations for disease association. The need of the hour is to curate these resources using standards for data exchange over the web as also using standard ontologies for data analysis across platforms.

Most of the mitochondrial diseases have rare occurrence in the population and hence are termed as ‘Rare diseases’. As of now there are nearly 7000 rare diseases reported worldwide arising from mutations in either nDNA or mtDNA. It is estimated that nearly 300 million people in the world are affected by rare diseases. In India alone there are nearly 70 million people diagnosed with rare diseases ( http://www.rarediseasesindia.org/), despite the fact that there are no standard diagnostic tests available for most rare diseases. In addition, the definition of rare diseases vary, for e.g., the European Union considers diseases to be rare when they affect no more than 5 in 10,000 people, while the United States of America (USA) consider a disease to be rare when affecting fewer than 200,000 people. In Asia, the threshold is 1 in 10,000. However, the prevalence of most mitochondrial diseases is not known ^9, 10^.

This review elaborates on the available resources, the bottlenecks in rare disease research and proposes innovative ways to address these challenges. There are many reviews discussing the challenges involved in establishing genotype-phenotype correlations with mitochondrial dysfunction given its involvement in multiple pathways in a spatio-temporal manner ^3, 5, 8, 11^. Here we review our current understanding of the rare disease initiatives and efforts that are ongoing globally along with specific focus on mitochondrial disease resources.

### Resources and challenges: ongoing efforts & translational pipelines

The first human DNA to be completely sequenced was the human mitochondrial DNA (16569 base pairs) in 1981 ^12, 13^. Given the relatively small size and absence of repeats, sequencing and assembling the mitochondrial human genome was not as challenging and difficult as sequencing the human nuclear genome using the Sanger Sequencing technology. However, mitochondrial genome sequencing has its own unique problems given the high mutation rate and high levels of heteroplasmy. Precise determination of the levels of heteroplasmy is crucial since the level of heteroplasmy determines both the penetrance and severity of expression of some mitochondrial diseases. The next generation sequencing (NGS) technologies like Virtual terminator sequencing (Illumina), Pyrosequencing (Roche) and SOLID have allowed to overcome these limitations by providing massive parallelization, high coverage, high accuracy as compared to Sanger sequencing. Specific protocols, including long range PCR with mitochondria specific primers, and algorithms for reference based and *de novo* assembly have been developed to sequence mitochondrial DNA using NGS technologies ^14^. NGS-based clinical targeted gene assay for the mitochondrial genome and 108 selected nuclear genes associated with mitochondrial disorders have also been designed to facilitate the analysis and understanding of nuclear and mitochondrial variations in mitochondrial diseases ^15^. These emerging technologies offer an excellent opportunity to further dissect the molecular basis of disease manifestation ^4^. With an increasing number of individuals that may be genetically screened across different populations, excellent datasets may be available to explore the genetic basis of disease. The genomics data along with clinical and biochemical profiles may also be used to identify disease biomarkers with high sensitivity and specificity, which is a major challenge in diagnosing mitochondrial dysfunction.

Over the years, a large number of web-based resources have been developed on various aspects of mitochondrial diseases, most of them focusing on the data from mtDNA. Some of these include, MitoMap, a database on human mitochondrial variation ^6^, MitoLSDB, the largest curated data on mtDNA variation with phenotype using LOVD ^16^, MitoCarta, a resource on mitochondrial proteins based on localization ^17^, MitoMiner, a mitochondrial protein identification system based on multiple evidences ^18, 19^, MitoBreak, a curated dataset on mtDNA rearrangements ^20^, HmtDB, an online resource for data on mitochondrial genome sequences annotated with population and variation data ^21^, Mitochondrial Database (mitoDB), the mitochondrial database on clinical features seen in mitochondrial diseases ^22^, to name a few. Analysis pipelines and platforms have also been developed, including the MtSNPscore which assesses the role of variation in context of disease association using a combined evidence approach ^23^, Mit-o-matic, an analysis pipeline for clinical evaluation of mitochondrial variations from the NGS datasets ^24^. More recently, the United Mitochondria Disease Foundation (UMDF) ( http://www.umdf.org/site/c.8qKOJ0MvF7LUG/b.7929671/k.BDF0/Home.htm) along with the National Institute of Child Health and Human Development (NICHD) ( http://www.nichd.nih.gov/Pages/index.aspx) launched the Mitochondrial Disease Sequence Data Resource (MSeqDR) Consortium. The goals of this consortium is to facilitate deposition, curation, annotation and integrated analysis of genomic data for mitochondrial diseases for clinical and research communities ^25^. The list of various mitochondrial resources may be seen in Table 1.

**Table 1.** Lists of mitochondrial and rare diseases resources. There are a large number of resources developed both for mitochondrial community and rare diseases community. The first section lists the resources available on mitochondrial diseases including Support and Advocacy groups ^*^, databases and analysis pipelines ^#^, research ^&^ and patient networks ^$^. The second section lists resources on rare diseases.

S. No	Name	Description/URL
Mitochondrial Disease Resources		
** a. Support and Advocacy**		
1.	International mito-patients (IMP)	IMP is a network of patient organization working on mitochondrial disease. http://www.mitopatients.org/index.html
2.	MitoAction	MitoAction is a support group which provides education and advocacy for mitochondrial diseases patients. http://www.mitoaction.org/
3.	United Mitochondrial Disease Foundation (UMDF) ^&^	Promotes research and education for the diagnosis, treatment and cure of mitochondrial disorders and to provide support to affected individuals and families http://www.umdf.org/
4.	The Children’s Mitochondrial Disease Network	The Children’s Mitochondrial Disease Network is an information and support group for patients with mitochondrial disorders. http://www.emdn-mitonet.co.uk/index.htm
5.	MitoCanada	Canada’s only patient advocacy organization for mitochondrial disease, dysfunction and mito-health. Active in communities across country, MitoCanada offers patient support and programs, research funding, public education and awareness campaigns, and advocacy. www.mitocanada.org
** b. Research**		
6.	Mitochondrial Research Guild (MRG)	MRG is an organization of Seattle Children's Hospital that works to raise awareness, promoting research and improving quality of medical care. http://www.nwmito-research.org/
7.	North American Mitochondrial Disease Consortium (NAMDC)	Works towards collecting information in mitochondrial disease patients in a clinical patient registry http://www.rarediseasesnetwork.org/namdc/index.htm
8.	The Mitochondrial Medicine Society (Mitosoc)	Mitosoc is a society for physicians, clinicians and researchers working for the better diagnosis, management and treatment of mitochondrial diseases. http://mitosoc.org/
9.	The Mitochondrial Research Society (MRS)	Organization for scientist and researchers working for the cure of mitochondrial diseases. http://www.mitoresearch.org/index.html
10.	The Rare Mitochondrial Disease Service for Adults and Children (RMDSAC)	RMDSAC is an information web resource for all aspects of mitochondrial disease. http://www.mitochondrialncg.nhs.uk/
** c. Database and Analysis Pipelines**		
11.	Human Mitochondrial Database (HmtDB)	An online web resource for population genetics and mitochondrial disease. http://www.hmtdb.uniba.it/hmdb/
12.	MitoBreak	MitoBreak is an online resource of curated datasets of mtDNA breakpoints. http://mitobreak.portugene.com/cgi-bin/Mitobreak_home.cgi
13.	MitoCarta	MitoCarta is Data inventory for genes for Human and Mouse which encodes proteins with strong support for mitochondrial localization. http://www.broadinstitute.org/pubs/MitoCarta/
14.	Mitochondrial Disease Database (MitoDB)	MitoDB is a database and analysis resource for mitochondrial disease. http://www.mitodb.com/
15.	Mitochondrial Disease Sequence Data Resource (MSeqDR)	MseqDR is a consortium for data capturing, integration, visualization and analysis of genomic data for mitochondrial diseases. https://mseqdr.org/index.php
16.	MitoLSDB	MitoLSDB is the most comprehensive web resource for mtDNA variations on LOVD platform. https://ab-openlab.csir.res.in/mitolsdb/home.php
17.	MITOMAP	Mitomap is a database of human mitochondrial genome. http://mitomap.org/MITOMAP
18.	Mit-o-matic	It is an analysis tool for finding clinical correlation of human MtDNA variations. http://genome.igib.res.in/mitomatic/index.html
19.	MitoMiner	An integrated data warehouse of proteomic data for mitochondria http://mitominer.mrc-mbu.cam.ac.uk/release-3.1/begin.do
20.	MitoVariome	MitoVariome is a database of human mitochondrial DNA variation. http://variome.kobic.re.kr/MitoVariome/index.jsp
21.	MtSNPscore	MtSNPscore is combined evidence based approach for prioritizing disease-associated variants. http://ab-openlab.csir.res.in/snpscore/
** d. Others**		
22.	Matchmaker Exchange	A federated platform to facilitate matching similar phenotypic and genotypic profiles (matchmaking) through standardized application programming interfaces (APIs) http://matchmakerexchange.org/
23.	Beacon Project	A web service for comparing genomic data maintaining data privacy http://ga4gh.org/#/beacon
24.	Global Alliance for Genomics and Health (GA4GH)	GA4GH is a coalition for effective and responsible data sharing for advancement in human health through genomics medicine. http://genomicsandhealth.org/
**Rare Disease Resources**		
1.	Genetic and Rare Disorder Information Center (GARD)	GARD is an information center for genetic and rare diseases. http://rarediseases.info.nih.gov/gard
2.	Global Genes	Global Genes is advocacy organization for rare disease patients. https://globalgenes.org/
3.	Global Rare Disease Patient Registry Data Repository (GRDR)	GRDR is a global data repository for patient information, clinical data and clinical trials data for researchers. https://grdr.ncats.nih.gov/
4.	International Rare Disease Research Consortium (IRDiRC)	IRDiRC is a consortium of researchers and organization working on diagnosis and therapies for rare diseases. http://www.irdirc.org/
5.	National Organization for Rare Disorders (NORD)	A non-profit organization offering services for identification, treatment and cure of rare disorders through advocacy, research and programs of education http://www.rarediseases.org/
6.	Orphanet	Orphanet is portal for rare diseases and orphan drugs. http://www.orpha.net/consor/cgi-bin/index.php?lng=EN
7.	Patients Like Me	A for-profit company providing platform for patients to share their condition and experience for the benefit of the larger patient population. http://www.patientslikeme.com/
8.	Rare Diseases Clinical Research Network (RDCRN)	Focuses on advancing medical research on rare diseases by facilitating collaboration, study enrollment and data sharing https://www.rarediseasesnetwork.org/
9.	Rare Diseases India	Rare Diseases India is knowledge portal driven by volunteers, students, patients, health care professionals and experts. http://www.rarediseasesindia.org/about
10.	The Office of Rare Diseases Research (ORDR)	ORDR is part of NCATS that supports research and provides research on rare disease. http://rarediseases.info.nih.gov/
11.	Therapeutics for Rare and Neglected Disease (TRND)	TRND is a NCATS program for stimulating new drug discovery and development for rare and neglected diseases. http://www.ncats.nih.gov/research/rare-diseases/trnd/trnd.html

Globally, attempts have also been made to systematically address the problem of rare diseases by establishing focused programs and consortia-based approaches. Therapeutics of Rare and Neglected Diseases (TRND) ( http://www.ncats.nih.gov/research/rare-diseases/trnd/trnd.html), a program led by the National Center for Advancing Translational Sciences (NCATS) ( http://www.ncats.nih.gov/), supports the development of potential treatments for rare and neglected diseases to first-in-human trials. This approach provides a de-risking strategy making the downstream development efforts commercially viable. TRND also supports the pre-clinical studies including medicinal chemistry optimization, drug metabolism and pharmacokinetics, toxicology formulation, and others studies required to file Investigational New Drug (IND) application for regulatory approvals. The other initiatives by NCATS for rare diseases include Office of Rare Disease Research (ORDR) ( http://www.orphadata.org/cgi-bin/inc/ordo_orphanet.inc.php) which coordinates a large number of collaborative research efforts towards rare diseases including support to institutes and centers, managing patient registry, human bio-specimen repository, to name a few major activities. Rare Diseases Clinical Research Network (RDCRN) ( http://rarediseases.info.nih.gov/research/pages/41/rare-diseases-clinical-research-network), from ORDR, focuses on advancing medical research on rare diseases by facilitating collaboration, study enrollment and data sharing. It also connects scientists from multiple disciplines across various clinical sites globally to work with patient advocacy groups. The North American Mitochondrial Disease Consortium (NAMDC) ( http://www.rarediseasesnetwork.org/namdc/), a part of RDCRN, specially works towards collecting information from mitochondrial disease patients in a clinical patient registry. In addition to periodically updating the patients on mitochondrial diseases, NAMDC also helps researchers to identify and recruit patients for future studies. Data generated as part of the various initiatives involving patient information are managed by the NCATS Global Research Patient Registry Data Repository (GRDR) ( http://www.ncats.nih.gov/research/rare-diseases/grdr/grdr.html). This is a web-based resource that aggregates de-identified patient information across many registries and provides a Globally Unique Identifier (GUID) to each patient data. GUID allows for patient follow-up across different registries, diseases, studies and countries and also ensures that clinical information is also mapped to bio-specimen datasets. Bridging interventional Development Gaps (BrIDs) ( http://www.ncats.nih.gov/research/rare-diseases/bridgs/bridgs.html) is a division of NCATS for pre-clinical innovation towards the development of new therapeutic agents both for common and rare diseases. A recent perspective on the NCATS TRND and BrIDGs programs highlights the role of team effort where academia, biotech and pharma industries, patient communities, advocacy groups, regulators, and government support, all are needed to navigate through the translational Valley of Death ^26^. National Organization for Rare Disorders (NORD) ( http://www.rarediseases.org/) is a non-profit organization with the aim to improve the lives of all people affected by rare diseases. The services offered by NORD include identification, treatment and cure of rare disorders through advocacy, research and programs of education. Similarly, Global genes is a non-profit organization for patient advocacy and work towards building awareness and providing connections and resources for rare disease patients ( http://globalgenes.org/). Similar efforts in the European subcontinent have led to the establishment of Orphanet, a consortium of 40 countries coordinated by the French INSERM team and which hosts a reference portal for rare diseases and orphan drugs. Orphanet hosts a directory of information of expert clinics, medical labs, clinical trials, patient organizations, etc. ( http://www.orpha.net/consor/cgi-bin/index.php). The joint effort of the European Commission and the NIH established IRDiRC, the International Rare Diseases Research Consortium, in 2011. This international consortium of researchers and organizations aims to deliver 200 therapies and means to diagnose most rare diseases by 2020, and as per their reports, the targets are being delivered ( http://www.irdirc.org/). The list of various rare diseases resources may be seen in Table 1.

As mentioned earlier, there are no clear estimates of the number of mitochondrial rare diseases. In order to get an approximation, the list of 6537 rare diseases was taken from Global genes website. To find out which rare disease is also a mitochondrial disease, a list of mitochondrial diseases was taken from the Mitochondrial Database (mitoDB) (51) ^22^. In addition, information on mitochondrial diseases is also referred from UMDF (41) and MITOMAP (51) ^6^. On comparing rare disease list with the mitochondrial diseases lists, 18 rare mitochondrial diseases were identified. It is important to mention here that since an exact match was performed we could have missed diseases for which abbreviation or synonyms are used. This also highlights that standard ontologies and terms are not systematically followed making data intractable for automated data analysis. We further checked the prevalence of these 18 rare diseases from the Rare Diseases India ( http://www.rarediseasesindia.org/) and Orphanet portals. As may be seen on Table 2, data are available for few diseases only.

**Table 2.** List of rare mitochondrial diseases. The table provides a list of diseases that cause mitochondrial dysfunction and are also reported rare diseases.

Rare Diseases	India		Europe	
	Per 100,000	Number of reported cases	Per 100,000	Number of reported cases
Acute megakaryoblastic leukemia	NA	NA	NA	NA
Barth syndrome	NA	NA	0.22	NA
Chronic progressive external ophthalmoplegia	NA	NA	NA	NA
Coenzyme Q10 deficiency	NA	NA	NA	NA
Familial bilateral striatal necrosis	NA	NA	NA	NA
Friedreich ataxia	NA	NA	2	NA
Infantile onset spinocerebellar ataxia	NA	NA	NA	24
Kearns Sayre syndrome	NA	NA	2	NA
Leber hereditary optic neuropathy	7	84000	1.5	NA
Leigh syndrome	3	36000	2.75**	NA
Maternally inherited Leigh syndrome	NA	NA	NA	NA
Pyruvate carboxylase deficiency	NA	NA	NA	NA
Pyruvate dehydrogenase deficiency	NA	NA	NA	NA
Pyruvate dehydrogenase phosphatase deficiency	NA	NA	NA	NA
Rett syndrome	4	48000	4	NA
Spastic paraplegia 7	NA	NA	NA	NA
Spinocerebellar ataxia 28	NA	NA	NA	NA
Sudden infant death syndrome	NA	NA	NA	NA

** indicates birth prevalence

### Crowdsourcing and crowdfunding rare diseases research and development

It is evident from a simple search in the clinical trials registry ( https://clinicaltrials.gov/) that only 0.2% of clinical trials ever done, ongoing, terminated or planned are for rare diseases. This clearly indicates that the major challenge for rare diseases research is the cost involved in research and development given the poor return on investment. It is also well known that clinical trials are prohibitively expensive even for common diseases where finding expert clinicians and acceptable number of patients is not as challenging as for rare diseases. These issues are addressed by de-risking research, developing platforms for sharing patient data, generating centralized patient registries and offering various incentives for making the development commercially viable. All these aspects have been discussed earlier. Here we discuss the various models that have been attempted to bridge the last mile of developing novel therapeutics.

One of the major challenges in the treatment of rare diseases is clinical trials patient recruitment. To overcome this challenge, patient advocacy groups and websites like PatientsLikeMe are turning out to be a major game changer. PatientsLikeMe is a patient powered research network that allows people to connect and share their experience with other people having the same disease or condition. PatientsLikeMe started its first online community for Amyotrophic Lateral Sclerosis (ALS) ^27– 29^ in 2006 where participants could ask specific questions about the treatment options and what to expect to fellow users ( http://www.patientslikeme.com/). In addition, the patients also got involved in experimenting with drugs that have not received regulatory approval. Thus data generated by these self-reporting platforms can be used to establish the efficacy and safety of a compound for rare diseases. The self-reported data help provide evidence to support or refute treatment outcomes. Despite several limitations of self-reported data, like unmeasured covariates, data reliability and controlled settings, these approaches are promising as has been shown with the quality of data gathered with smartphone games which were comparable to data obtained from controlled laboratory environments ^30, 31^. In 2011, the company expanded its scope and allowed any patient with any condition to join the community. Currently there are more than 300,000 members registered on the PatientLikeMe community with more than 2000 health conditions ( http://en.wikipedia.org/wiki/PatientsLikeMe). Besides, interactions of the patient advocates from the rare diseases group with major regulatory bodies has led to expedite review and approval process for rare disease treatments ( http://www.forbes.com/sites/medidata/2014/09/25/rare-disease-patient-voices-bring-change-to-the-clinical-trials-process/).

Using another interesting strategy, Pfizer Inc. used a web-based interactive platform to evaluate the efficacy of a drug (tolterodine tartrate extended release capsules) to treat overacting bladder. This project called REMOTE is a phase IV trial under an Investigational New Drug (IND) application ^32^. The participants for the trial were recruited through an interactive web-based platform from one clinical site overseen by physician ^33^. Despite poor patient participation, the study reports that the trial outcomes are consistent with the results from the conventional trial. The observations from REMOTE is a learning experience for the trial community on the challenges involved at various levels from patient understanding to technical issues. These learnings are used to conduct another trial in Europe, REMOTE2.0, to overcome the bottlenecks faced with the first attempt. These initiatives are of immense significance in establishing the robustness of trial strategies where patient recruitment is a challenge. These mobile technologies also allow patients with complex disabilities to participate in trials. The mobile technologies in form of wearable sensors also make the trial monitoring and patient diagnosis at different time points more affordable ^34^. Subsequent efforts to share patient data also have a positive impact on patient recruitment.

It is also proposed that the N-of-1 trial method may be used to evaluate new treatments. Under this model a single patient is the entire trial and the treatment outcome is measured at different time points for pre-treatment, treatment duration and post-treatment ^35^. If the treatment outcome is measurable and the patient is cured, the treatment may be considered effective.

Thus, the crowdsourcing strategy is an attractive tool for patient recruitment as it is affordable and time effective. Likewise, crowdfunding is also proposed as a viable option for financing various aspects of rare diseases research. Crowdfunding initiatives not only generate awareness about rare diseases but also lead to funding for supporting research activities. One such example is the ALS Ice Bucket Challenge leading to 1.2 million videos on Facebook and 2.2 million tweets in a matter of 3 months, which raised over $100 million of donations ( http://en.wikipedia.org/wiki/Ice_Bucket_Challenge). Through innovative ideas like ALS Ice Bucket Challenge it is possible to raise funds and also increase public awareness about the rare diseases. The Rare Genomics Institute (RGI) ( http://raregenomics.org/) is an international non-profit providing expert network and an online crowdfunding mechanism to assist families pursue personalized research projects for diseases which would not be studied otherwise. RGI offers sequencing facilities for diagnosis, expert guidance on sequencing and systematic interpretation of the data generated. A recently published success story is that of a 3-year old girl showing symptoms of involuntary eye movement, small-sized head, involuntary muscle contraction, development delay and progressive decline. Whole exome sequencing of the family revealed a novel mutation that causes mental retardation and severe developmental delays. Through the RGI platform, $5000 was raised in 50 days to carry out the genome sequencing in the girl and her family, which led to identification of the novel mutation ^36^. These examples underline the potential of crowdfunding in addressing scientifically challenging and socially important problems.

With the increasing disease burden, the need of the hour is to establish new models for ensuring that clinical trials are sustainable and transparent. Clinical trials are generally demanding on both human and other resources. It is not possible to apply the conventional methods to rare disease trials given the shear costs involved. To overcome these bottlenecks, efforts are being made lately to reverse the approach of rare disease patient recruitments for clinical trials by taking the trials to the patients and not *vice versa*. This approach demands establishing patient-centric sites which is again challenging given their extremely sparse distribution. Telemetry innovations may offer solutions to this problem, which allows health monitoring of the patients remotely. This model is not just suitable for rare diseases but is also applicable to common diseases trials given that many clinical trial sites never recruit patients ( http://www.clinicalleader.com/doc/how-rare-disease-know-how-can-shape-big-pharma-clinical-trials-0001).

### Open data sharing and analysis platforms as drivers for innovation

Open access platforms which allow for data integration and exchange of ideas to facilitate the process of bringing new therapeutic interventions and diagnostic methods to the market are needed to drive sustained research and development for rare diseases. It is also imperative to synchronize the efforts at all levels of research, diagnosis and regulatory guidelines for evidence-based clinical decision-making. As part of the platform, it is also important to involve regulatory agencies from very early on in the discovery pipeline so that the progression through development and approvals is quicker and more affordable, which is the bottleneck with rare diseases. One strategy which seems to work well is to apply approaches of drug repurposing where it is crucial to understand the mechanism of disease manifestation and subsequently applying various modeling approaches to find new indications of existing drugs in the market ( http://www.fda.gov/ForIndustry/DevelopingProductsforRareDiseasesConditions/HowtoapplyforOrphanProductDesignation/ucm216147.htm). This strategy will be even more successful where one can systematically list the phenotypic parameters provided by patient groups in close collaboration with clinicians.

**Figure 1.**
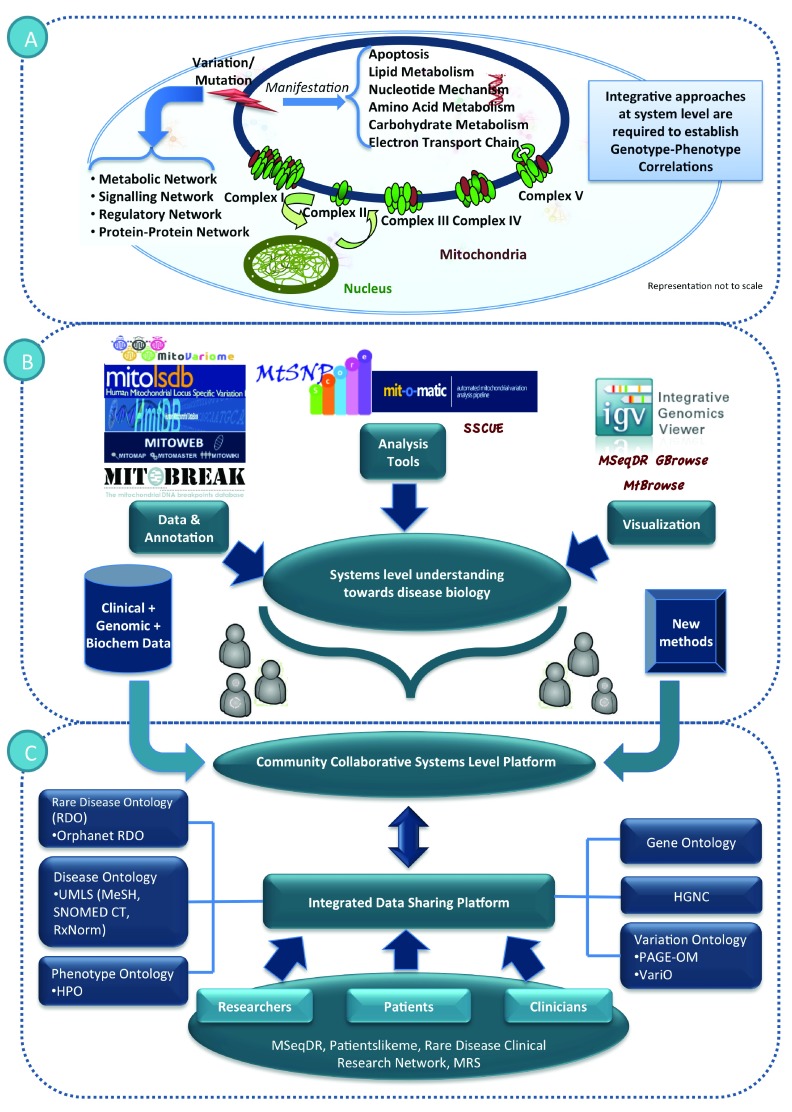
( **A**) This section describes the challenges involved in dissecting the impact of genomic variation in disease association. As shown, mitochondria are involved in a large number of cellular processes including energy metabolism. Genes encoded by mitochondrial and nuclear genomes carry out these functions. The protein subunits of the complexes of the electron transport chain are encoded both by mtDNA (red) and nDNA (green). In order to understand the complex genotype-phenotype correlation it is imperative to identify the molecular interactions at systems level (protein-protein interaction network, metabolic network, signaling network, regulatory network). It is important to curate the information needed to generate these networks from literature and existing resources. ( **B**) The resources currently available for mitochondrial dysfunction include databases, web servers and analysis pipelines as shown. For generating systems level models, these resources may be integrated systematically. ( **C**) There are various ongoing efforts involving researchers, clinicians and or patient groups. It is proposed that a community collaborative open access platform is a must to interface these communities. In order to establish such a platform that allows geographically different communities to work together, globally accepted ontologies in a language independent representation are needed.

There is a need for innovation in technology that allows for interfacing the different stakeholders, namely, researchers, clinicians and patient groups. Such resources will go a long way in future to allow patients to perform diagnostic procedures with greater accuracy and help clinicians identify the best possible route for therapeutic interventions. This is only possible if the participating members use standard terms to share the data. Various ontologies have been developed over years for capturing function of genes like the Gene Ontology ^37^, PAGE-OM ^38^ and VariO ^39^ for capturing features of variation, UMLS which includes MeSH ^40^, RxNORM ^41^ and SNOMED CT ^42^ for capturing clinical details and Human Phenotype Ontology ^43^ for phenotype data, to name a few. These ontologies are meant to function as a common set of vocabulary that needs to be shared on a community collaborative platform. The semantic network of these terms will allow deciphering and interpreting novel patterns in understanding disease biology. Such a semantic-based platform will be amenable for plugging in data and new methods with increasing understanding of disease biology. It will also ensure that the data generated as part of negative results are also shared systematically with scientific community. Orphanet Rare Disease Ontology (ORDO) ( http://www.orphadata.org/cgi-bin/inc/ordo_orphanet.inc.php) is an effort to achieve these goals. ORDO offers definitions, classification of rare diseases, gene-disease relations, epidemiological data and connections with other terminologies like MeSH, UMLS ( http://www.nlm.nih.gov/research/umls/), OMIM ^44^, UniProtKB ^45^, HGNC ^46^, ensembl ^47^, Reactome ^48^, etc. It is imperative that existing resources utilize these ontologies for data sharing allowing for data interoperability across different platforms. Figure 1 illustrates the scope of the proposed integrative platform describing the scientific challenges involved in establishing genotype-phenotype correlations and how the existing resources and community collaborations may be converged towards a systems level understanding of the disease biology.

### What’s new?

‘Internet of DNA’ is listed as one of the top 15 breakthrough technologies of 2015 ( http://www.technologyreview.com/featuredstory/535016/internet-of-dna/?utm_campaign=newsletters&utm_source=newsletter-weekly-biomedicine&utm_medium=email&utm_content=20150224). The technology needed to harness the power of genomics lies in comparing the genetic information from a large number of individuals with medical records. This currently is a huge challenge, partly because of the technical reasons of moving petabytes of data across different labs, but especially due to the privacy issues surrounding patient information. Both these issues should be addressed to ensure that the ever-increasing amount of genomic and clinical data piling up in laboratories and hospitals are utilized optimally. Upcoming initiatives like the MatchMaker Exchange are aiming to bring the genotype and phenotype data together on a common platform ( http://matchmakerexchange.org/). Global Alliance for Genomics and Health also known as GA4GH is an organization which provides protocols, APIs and file formats for effective and responsible sharing of genomic and clinical data ( http://genomicsandhealth.org/). The organization goal is to overcome challenges likes ethics and privacy involved with sharing of genomics data and to accelerate the potential of genomic medicine for advancement of human health. As discussed before, the need of the hour is to let the patients decide on who will access their data and how these may be used. This is only possible if the information generated using patient samples is made available in real-time. It is also important that other components of this major collaborative strategy are also part of a community platform that allows for gated access to patient data with patients deciding how and with whom their data should be shared.

In the changing paradigms of disease treatment, a very recent approval is made by Britain on Mitochondrial donation ( http://www.theguardian.com/politics/2015/feb/24/uk-house-of-lords-approves-conception-of-three-person-babies). In this approach *in vitro* fertilization (IVF) technique is used with biological material coming from three parents, mother and father (contributing 98.8% genetic material) and a female donor (contributing 0.2% genetic material). This three-parent IVF approval in UK has received mixed reactions and only time will address the concern of the long-term implications of the same.

## Conclusions

Rare diseases affect over 300 million people globally, however the true burden of these diseases on human health remains to be determined. Rare genetic variants are disease causing and lead to a personalized disease manifestation. Thus, it is time to review the disease definition considering both the molecular mechanisms involved and environmental factors leading to differential phenotypes. This will allow for a better understanding of both rare and common diseases. On the other end, a paradigm shift in drug discovery and development is also needed to translate the effort in understanding disease mechanisms to identify potential therapeutic routes. Newer models and platforms that allow involvement of patient communities in research and development is also expected to offer solutions to patients suffering from rare diseases who may then benefit from appropriate treatment options. Community collaborative approaches for research and funding offer an unprecedented opportunity for making new discoveries and translating to therapeutic interventions.
