# Supplementary material for: Resources, challenges and way forward in rare mitochondrial diseases research
Source: F1000Res. 2015 Aug 13;4:70. Originally published 2015 Mar 16. [Version 2] doi: 10.12688/f1000research.6208.2 (PMC4490798; doi:10.12688/f1000research.6208.2)
Supplement: Peer review history

# Peer review: Referee response for version 2

**DOI:** 10.5256/f1000research.7458.r9986

**Published:** 2015-08-14

**Authors:** Vanniarajan Ayyasamy

**Affiliations:** 1 Department of Molecular Genetics, Aravind Medical Research Foundation, Madurai, Tamil Nadu, India

The authors had done a thorough compilation of all the possible resources for mitochondrial research and it will be a good starting point for all researchers

I have read this submission. I believe that I have an appropriate level of expertise to confirm that it is of an acceptable scientific standard.

# Peer review: Referee response for version 1

**DOI:** 10.5256/f1000research.6657.r9177

**Published:** 2015-07-01

**Authors:** Vanniarajan Ayyasamy

**Affiliations:** 1 Department of Molecular Genetics, Aravind Medical Research Foundation, Madurai, Tamil Nadu, India

This article really serves as a good resource for researchers, clinicians and also patients.

I would suggest the authors to compare the available databases of the mitochondrial research and may provide a better summary.

I have read this submission. I believe that I have an appropriate level of expertise to confirm that it is of an acceptable scientific standard.

# Response

**Published:** 2015-08-07

**Authors:** Anshu Bhardwaj

**Affiliations:** Open Source Drug Discovery Unit, Council of Scientific and Industrial Research, India

We thank the reviewer for encouraging comments. The scope of the current review was to provide an end-to-end view of the resources, challenges and way forward for addressing translational challenges in mitochondrial diseases. The comparison of databases of the mitochondrial research needs a separate review as the parameters for comparison need to be comprehensive and the information related to each resource should be discussed in details. The authors are working on the same and will share the details as soon as it is ready.

# Peer review: Referee response for version 1

**DOI:** 10.5256/f1000research.6657.r8964

**Published:** 2015-06-26

**Authors:** Xin Qi

**Affiliations:** 1 Center of Mitochondrial Disease, School of Medicine, Case Western Reserve University, Cleveland, OH, USA

This is a very good and informative review paper, which summarized nicely on the resources on research of mitochondrial diseases ranging from clinical setting to basic science.

I have read this submission. I believe that I have an appropriate level of expertise to confirm that it is of an acceptable scientific standard.
